# Draft Genome Sequence of Vancomycin-Heteroresistant *Staphylococcus epidermidis* Strain UC7032, Isolated from Food

**DOI:** 10.1128/genomeA.00709-13

**Published:** 2013-09-26

**Authors:** Simona Gazzola, Ester Pietta, Daniela Bassi, Cecilia Fontana, Edoardo Puglisi, Fabrizio Cappa, Pier Sandro Cocconcelli

**Affiliations:** Istituto di Microbiologia, Università Cattolica del Sacro Cuore, Piacenza, Cremona, Italy

## Abstract

*Staphylococcus epidermidis* strain UC7032 was isolated from ready-to-eat cured meat and is heteroresistant to glycopeptide antibiotics. The draft whole-genome analysis revealed that this strain shows common characteristics typical of strains that are involved in nosocomial infections.

## GENOME ANNOUNCEMENT

Coagulase-negative staphylococci, a common cause of human and animal infections, are frequently isolated from food, where they play a role in fermentation processes.

*Staphylococcus epidermidis* UC7032, isolated from cured pork, is a vancomycin-heteroresistant strain (1) and is able to form a biofilm-like structure in the presence of antibiotics or in food.

In this study, shotgun sequencing of strain *S. epidermidis* UC7032 was performed using an Illumina HiSeq 1000 sequencing system, provided by the Functional Genomic Centre of the Scientific and Technological Department, University of Verona. The high-quality filtered reads were assembled using Velvet software (version 1.1.04) (2), and 155 contigs were annotated by the RAST server (3). The draft genome sequence of food isolate UC7032 consists of 2,496,853 bp, with a G+C content of 31.9%, 2,360 putative coding sequences (CDSs), 27 predicted RNAs, and 371 subsystems.

Genome analysis showed the presence of staphylococcal enterotoxin C and K genes (*sec* and *sek*, respectively) and genes coding for proteins with adhesive functions, including those involved in the first phase of biofilm formation (*atlE*) and in catheter-associated infections (*fbe*) and the Bap homologue biofilm-associated protein Bhp (*bhp*). The strain UC7032 is classified in *agr* group II and harbors a recently identified family of proinflammatory peptides, the phenol-soluble modulins (PMSs), which have multiple functions in biofilm development and in evasion of the immune system (4). Furthermore, this strain carries the *sepA* and *sspA* genes, which are involved in the degradation of fibrinogen and are probably responsible for tissue damage and proteolysis of the biofilm matrix protein. Other virulence factors were found, such as *dtlABCD* (d-alanylation of teichoic acids), *mprF* (phosphatidylglycerol lysyltransferase), and *graR* and *graS*, which are involved in the Aps system (5). This food strain is classified as an arginine catabolic mobile element (ACME) type II allotype because it showed the presence of the *arc* gene cluster but not the *opp3* cluster (oligopeptide permease system) (6). No evidence of clustered regularly interspaced short palindromic repeat (CRISPR)-associated protein genes *cas1* and *cas2* was found. The genome analysis also showed the presence of genes involved in fluoroquinolone resistance (*parA*, *parC*, *gyrA*, *gyrB*), two genes of a mercury resistance operon (*merA* and *merB*), two putative plasmids (3,037 bp and 43,888 bp), and an incomplete prophage.

### Nucleotide sequence accession number.

The whole-genome shotgun project has been deposited at GenBank under the accession no. ARWU00000000. The version described in this paper is the first version.
